# Evaluation of prostate cancer antigen 3 for detecting prostate cancer: a systematic review and meta-analysis

**DOI:** 10.1038/srep25776

**Published:** 2016-05-10

**Authors:** Yong Cui, Wenzhou Cao, Quan Li, Hua Shen, Chao Liu, Junpeng Deng, Jiangfeng Xu, Qiang Shao

**Affiliations:** 1 Department of Urology, Suzhou Municipal Hospital, Nanjing Medical University Affiliated Suzhou Hospital, Suzhou, 215001, P.R. China; 2 Center for Cancer Genomics, Wake Forest University School of Medicine, Winston-Salem, NC 27157, USA

## Abstract

Previous studies indicate that prostate cancer antigen 3 (PCA3) is highly expressed in prostatic tumors. However, its clinical value has not been characterized. The aim of this study was to investigate the clinical value of the urine PCA3 test in the diagnosis of prostate cancer by pooling the published data. Clinical trials utilizing the urine PCA3 test for diagnosing prostate cancer were retrieved from PubMed and Embase. A total of 46 clinical trials including 12,295 subjects were included in this meta-analysis. The pooled sensitivity, specificity, positive likelihood ratio (+LR), negative likelihood ratio (−LR), diagnostic odds ratio (DOR) and area under the curve (AUC) were 0.65 (95% confidence interval [CI]: 0.63–0.66), 0.73 (95% CI: 0.72–0.74), 2.23 (95% CI: 1.91–2.62), 0.48 (95% CI: 0.44–0.52), 5.31 (95% CI: 4.19–6.73) and 0.75 (95% CI: 0.74–0.77), respectively. In conclusion, the urine PCA3 test has acceptable sensitivity and specificity for the diagnosis of prostate cancer and can be used as a non-invasive method for that purpose.

Prostate cancer is the most common cancer among men living in Western nations and now the most common malignancy (with a prevalence near that of bladder cancer) seen in urological clinics in China^1^. In the USA, prostate cancer is the most frequently diagnosed cancer and the second leading cause of cancer-related death in men, with an estimated 233,000 new cases and 29,480 deaths in 2014^2^. In clinical practice, serum prostate-specific antigen (PSA), digital rectal examination (DRE), transrectal ultrasound, and biopsy are widely used for early detection. Although PSA improved prostate cancer detection in the early “PSA era,” it has many limitations, especially when PSA values are 4–10 ng/ml (the “gray zone”).

In 1999, Bussemakers *et al*. identified the DD3 gene (later also known as prostate cancer antigen 3, or PCA3), which is highly expressed in prostatic tumors^3^. A new diagnostic method uses polymerase chain reaction (PCR) to detect the over-expression of PCA3 mRNA in urine. This non-invasive urine biomarker has been evaluated in many clinical studies, in several of which it was combined with other markers to improve the diagnostic accuracy in order to further rule out aggressive cancer at biopsy. In 2012, the US Food and Drug Administration (FDA) approved the PROGENSA PCA3 assay, the first molecular test to help determine the need for repeat prostate biopsies in men with a previous negative biopsy (U.S. Food and Drug Administration Summary of Safety and Effectiveness Data: PROGENSA PCA3 Assay, 2012. Available at www.accessdata.fda.gov/cdrh_docs/pdf10/P100033b.pdf&U.S. Food and Drug Administration Medical Devices: PROGENSA PCA3 Assay, 2012. Available at www.fda.gov/MedicalDevices/ProductsandMedicalProcedures/DeviceApprovalsandClearances/Recently-ApprovedDevices/ucm294907.htm).

We consider the current clinical evidence in investigating the diagnostic value of PCA3 in prostate cancer with both initial and repeat biopsy.

## Methods

This meta-analysis was performed in accordance with PRISMA^4^ guidelines, which prefer reporting items from systematic reviews and meta-analyses.

### Search strategy and study selection

A comprehensive, computerized literature search was performed in PubMed and Embase for work published through December 2014 using a combination of the following key words: [“prostatic neoplasm” or “prostate cancer”] AND [“PCA3” or “prostate cancer antigen3” or “dd3” or “upm3” or “aptima pca3”] AND [“diagnosis” or “sensitivity and specificity”]. Then, the reference sections of the identified publications were searched to identify additional potentially relevant articles. Studies included in our meta-analysis had to meet the following criteria: (1) case-control or cohort design; (2) diagnostic test using PCA3 itself or in combination with other biomarkers; and (3) prostate biopsy as the gold standard.

### Data extraction and quality assessment

Data were extracted independently by 2 authors (Y.C. and C.L.) and then crosschecked. For each study, the following information was collected: last name of the first author, publication year, study design and ethnicity, age, PSA, sample size and the values of true positive (TP), false positive (FP), false negative (FN), true negative (TN), and area under the curve (AUC) (with 95% CI) if available. When more than one article was published using the same population, we selected the most recent or most informative report. Disagreements between the two authors were resolved by consensus. The quality of the selected studies was assessed using quality assessment of diagnostic accuracy studies (QUADAS)^5^. The QUADAS tool consists of a set of 14 questions, each of which is scored as yes, no, or unclear.

### Statistical analysis

For each study, 2 × 2 tables for each test with TP, FP, FN, and TN results were extracted from the original scientific articles. Pooled estimates of sensitivity (Se) and specificity (Sp) and their 95% confidence intervals were calculated as the main outcome measures. Forest plots were used, and methodological heterogeneity was assessed during selection.

The threshold effect is a characteristic source of heterogeneity in the meta-analysis of diagnostic tests and arises when the included studies use different cut-off points to define a positive result of a diagnostic test. The analysis of the diagnostic threshold was assessed through the receiver operating characteristic (ROC) plane and Spearman’s correlation coefficient The ROC plane is the graphic representation of the pairs of Se and Sp, and it characteristically shows a curvilinear pattern if the threshold effect exists. Statistical heterogeneity was measured using the χ^**2**^ test and *I^2^* scores. The *I^2^* score was used as a measure of the inconsistency between studies in the meta-analysis and was interpreted as low (25–50%), moderate (51–75%), or high (>75%).

Data were analyzed using the statistical software package Metadisc, version 1.4. The results were synthesized and represented graphically in a forest plot. If heterogeneity was found, the meta-analysis was performed using a random effects model. If there was evidence of the threshold effect, the studies were combined to create a summarized ROC curve (SROC), to calculate an additional measurement of the accuracy of the technique (Q^*^) and to obtain the AUC.

## Results

**Figure 1:**
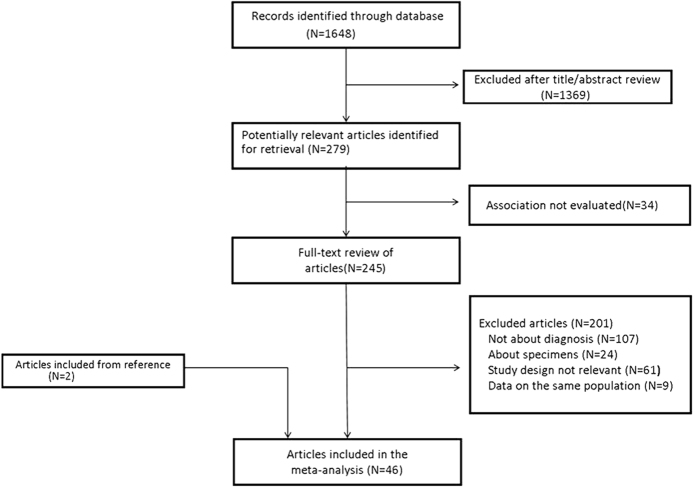
Literature search.

A total of 1,648 relevant references were obtained in our systematic search. The results and study selection process are shown in Fig. 1. There were 245 articles requiring full-text review, and 46 studies were included in the meta-analysis^6^,^7^,^8^,^9^,^10^,^11^,^12^,^13^,^14^,^15^,^16^,^17^,^18^,^19^,^20^,^21^,^22^,^23^,^24^,^25^,^26^,^27^,^28^,^29^,^30^,^31^,^32^,^33^,^34^,^35^,^36^,^37^,^38^,^39^,^40^,^41^,^42^,^43^,^44^,^45^,^46^,^47^,^48^,^49^,^50^,^51^. In addition, one study (by Hensen *et al*.^52^) that focused on combined initial prostate cancer biopsy in a North American and European multi-center cohort that overlapped in two studies^13^,^34^ was included in the stratified analysis of initial biopsy. Additionally, the study by Scattoni *et al*.^48^ included initial and repeat biopsy groups that were treated as two data sets. The quality of the selected studies on diagnostic testing was moderate to high according to the QUADAS scale (Table 1).

Based on the studies described above, we retrieved data from 12,295 patients with PCA3 test results and prostate biopsy, of whom 4,225 were diagnosed with prostate cancer. All studies presented the sensitivity, specificity, and cut-off points (25 studies had a cut-off of PCA3 = 35), and most studies presented the ROC curve (Supplementary Table 1). Among the 46 trials, most were performed in the U.S. and Europe; 5 were performed in Asia (Table 1).

**Figure 2:**
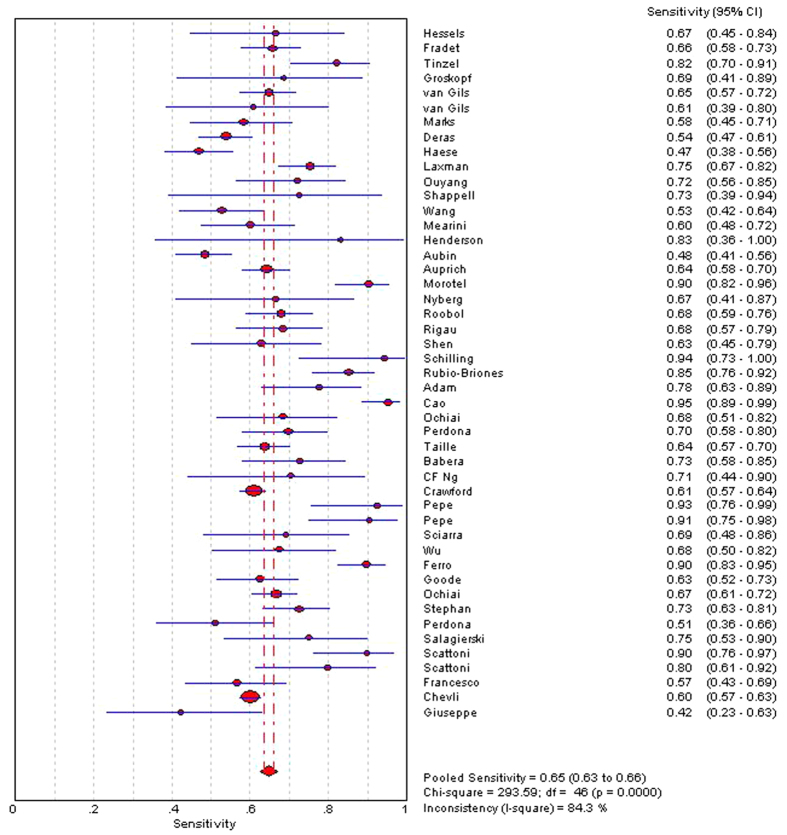
Forest plot of pooled sensitivity.

**Figure 3:**
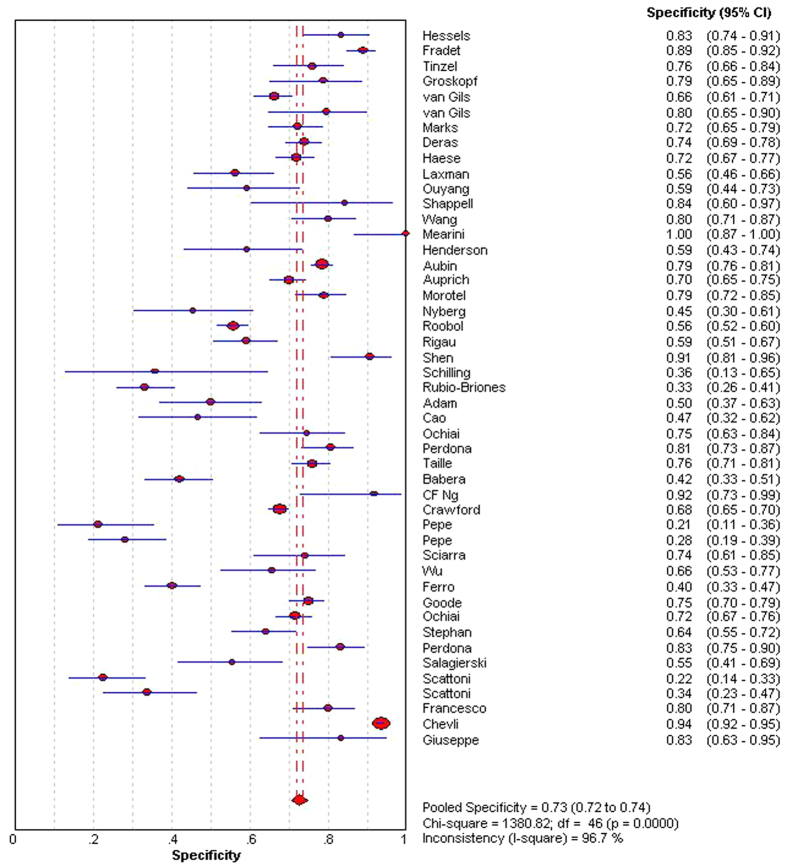
Forest plot of pooled specificity.

**Figure 4:**
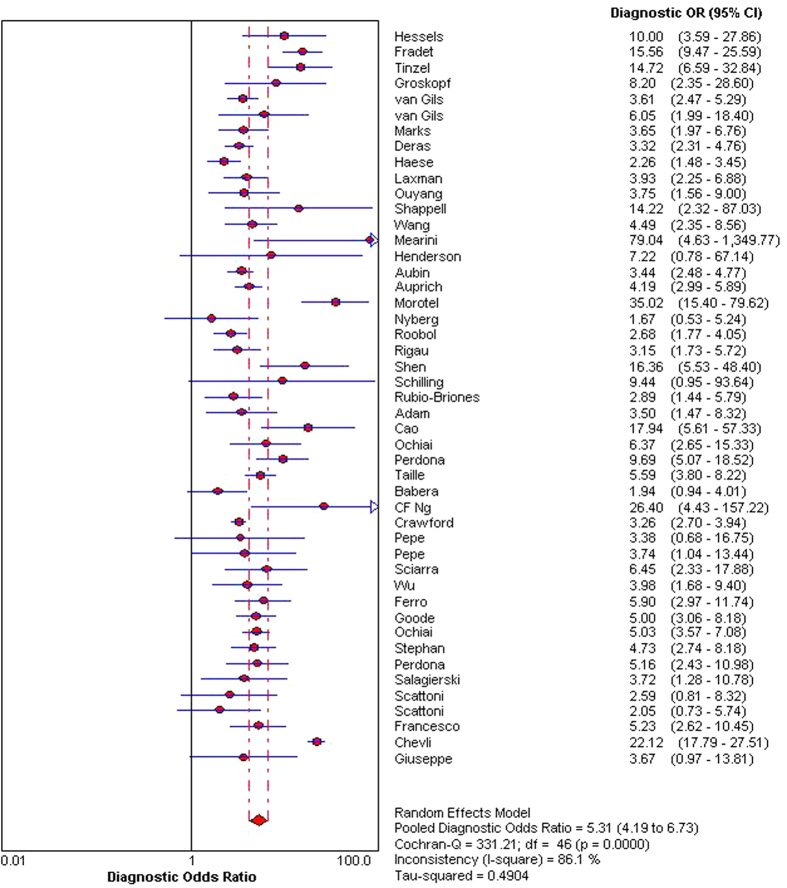
Forest plot of pooled diagnostic OR.

**Figure 5:**
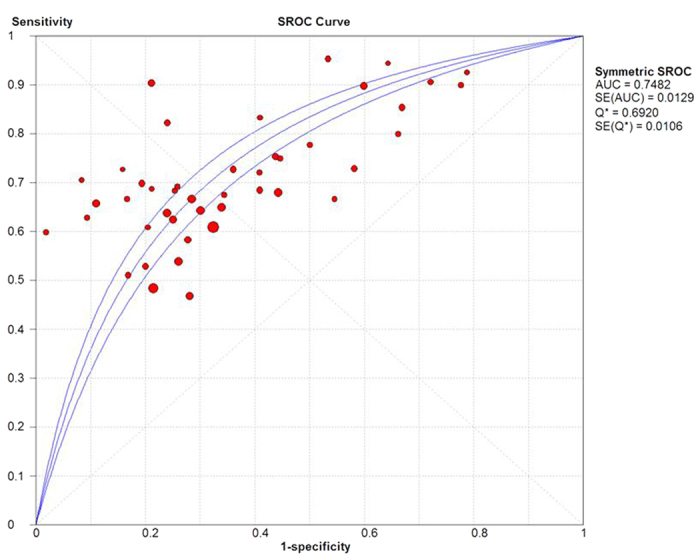
SROC curve.

The indices of diagnostic validity obtained from the 2 × 2 tables showed that sensitivity ranged from 46.9% to 95%, and specificity ranged from 21.6% to 100%. In the 40 articles that presented the AUC, it ranged from 0.57 to 0.85. A meta-analysis was conducted using the 46 articles mentioned above. The ROC space showed a curvilinear trend, and Spearman’s correlation coefficient was 0.612 (P < 0.001), which suggests the existence of a threshold. There was a high degree of heterogeneity in sensitivity (χ2 = 271.39, P < 0.001), specificity (χ2 = 735.87, P < 0.001), and diagnostic OR (Cochran-Q = 137.22, P < 0.001); consequently, the diagnostic indices were calculated using a random effects model. Using a forest plot, the overall sensitivity, specificity, positive likelihood ratio , negative likelihood ratio and diagnostic OR were 0.65 (95% CI 0.63–0.66) (Fig. 2), 0.73 (95% CI 0.72–0.74) (Fig. 3), 2.23 (95% CI: 1.91–2.62), 0.48 (95% CI: 0.44–0.52) and 5.31 (95% CI: 4.19–6.73) (Fig. 4), respectively. We used a summary SROC to aggregate data and obtained a symmetrical curve with an AUC of 0.748 (Fig. 5) that represented the technique’s diagnostic performance.

## Discussion

The clinical value for prostatic cancer diagnoses was not conclusive. According to the European Association of Urology and the National Comprehensive Cancer Network guidelines, the need for prostate biopsy should be determined on the basis of PSA and/or a suspicious DRE^53^,^54^. However, serum PSA levels can be elevated in benign conditions, and only 25% of men who are clinically suspected of having PCa will have a positive biopsy^55^,^56^. Thus, other biomarkers with high sensitivity and specificity are needed for screening.

The PCA3 gene was mapped to chromosome 9q21–22, in antisense orientation within intron 6 of the Prune homolog 2 gene (PRUNE2 or BMCC1), spanning a region of approximately 25 kb^57^,^58^. Ferreira *et al*. found that PCA3 may modulate PCa cell survival, and PCA3 expression is androgen-regulated via activation of AR-mediated signaling^59^. PCA3 is a non-coding, prostate-specific mRNA that is highly over-expressed in 95% of PCa cells, with a median 66-fold up-regulation compared with adjacent non-neoplastic cells^60^. Because PCA3 does not encode a protein, the only molecule that can be tested is the mRNA; PCA3 mRNA can be measured in urine sediment after DRE. A PCA3 score is the ratio of PCA3 mRNA to PSA mRNA multiplied by 1,000. Although we currently have a good understanding of the role of PCA3 in tumor genes and tissues, the picture is incomplete.

Several studies have indicated that the PCA3 test is useful in reducing the number of negative biopsies^34^,^39^,^51^, and more recently the FDA approved the PROGENSA PCA3 assay as a new test for prostate cancer.

Recent advances have included biomarkers such as HPG-1, AMACR, STAMP1, TMPRSS2, ERG, PHI, and P2PSA^31^,^42^,^48^,^61^,^62^,^63^. Some studies have assessed their efficacy by detecting these markers alone or in combination. Although several biomarkers may have specificity that is the same as or higher than that of PCA3, the non-invasive nature of the urine PCA3 test, which is performed after prostate massage, and its good diagnostic performance may make the PCA3 test a better choice for prostate cancer screening.

The current meta-analysis shows the clinical usefulness of this tumor marker in detecting prostate cancer with initial or repeat biopsy. We synthesized the current knowledge about early diagnosis of prostate cancer with PCA3 determination in urine samples. According to the data from 46 studies analyzed, specificity is 0.65 (range 47–95%), which is not adequate; sensitivity is 0.73, which is somewhat lower, and had a minimum value of 21%. The AUC of 0.75 obtained in the SROC curve suggests acceptable performance of the diagnostic test.

In the stratified analysis, a forest plot of initial biopsy showed overall sensitivity and specificity of 0.65 (95% CI 0.63–0.67) and 0.82 (95% CI 0.81–0.83), respectively, and a symmetrical curve with an AUC of 0.80 (95% CI 0.78–0.82) (Table 2). With repeat biopsy, these values dropped to 0.58 (95% CI 0.55–0.62), 0.69 (95% CI 0.67–0.71), and 0.68 (95% CI 0.67–0.70) (Table 2), respectively. Mixed biopsy showed values of 0.66 (95% CI 0.64–0.68), 0.68 (95% CI 0.67–0.69), and 0.75 (95% CI 0.74–0.76) (Table 2), respectively. These results suggest that PCA3 is potentially more suitable for initial prostate biopsy than repeat prostate biopsy. When we stratified the studies by PCA3 cut-off value, the overall sensitivity, specificity, and symmetrical curve AUC values were 0.63 (95% CI 0.62–0.65), 0.74 (95% CI 0.73–0.75), 0.74 (95% CI 0.73–0.76), respectively, for studies with a cut-off value ≠ 35 (Table 2) and 0.70 (95% CI 0.68–0.73), 0.67 (95% CI 0.65–0.69), and 0.77 (95% CI 0.75–0.79), respectively, for studies with a cut-off value = 35 (Table 2). Although the latter group showed better diagnostic performance, it has a greater range and more variable outcomes. This observation supports a cut-off of 35 for the standard value and clinical practice of many institutions. Comparing study designs, the overall sensitivity, specificity, and symmetrical curve AUC values were 0.63 (95% CI 0.61–0.66), 0.88 (95% CI 0.87–0.90), and 0.82 (95% CI 0.79–0.85) (Table 2), respectively, for case-control studies and 0.65 (95% CI 0.63–0.66), 0.73 (95% CI 0.72–0.74), and 0.75 (95% CI 0.74–0.76) (Table 2), respectively, for prospective studies. Because case-control studies typically enroll fewer patients and have greater heterogeneity, their quality is not as good as that of prospective studies.

Our meta-analysis has some limitations. First, the study numbers and the heterogeneity of their approaches influence the accuracy. Although the gold standard (biopsy) was used in all studies, the patient selection, lack of blinding, and different PCA3 cut-off values caused heterogeneity. Second, a potential publication bias may exist, although we tried to avoid this bias by expanding our searches in different databases and by conducting rigorous screening for studies. We evaluated the quality of the articles according to the QUADAS questionnaire. The quality of the studies in terms of diagnostic testing was moderate to high. The relatively small number of trials included in this meta-analysis and significant heterogeneity across the studies may make our conclusion conservative.

According to the current meta-analysis, the PCA3 test shows good diagnostic performance. However, it requires further exploration in well-designed and appropriately-powered trials to determine intermediate and long-term outcomes. Long-term observational studies of health outcomes are also subject to biases.

## Additional Information

**How to cite this article**: Cui, Y. *et al*. Evaluation of prostate cancer antigen 3 for detecting prostate cancer: a systematic review and meta-analysis. *Sci. Rep*. **6**, 25776; doi: 10.1038/srep25776 (2016).

## Supplementary Material

Supplementary Information

## Figures and Tables

**Table 1:** Methodological quality of the 46 studies according to the QUADAS questionnaire.

Study	Year	Country/region	Patients		Test				Result	
Author			Patients are representative of the question	Selection criteria described	Biopsy is performed in all patients	PCA3 assay described	Selection of controls	Number for cores per biopsy ≥10	Blinded gold standard interpretation	Cut-off reported
Hessels	2003	Netherlands/Eur	Yes	Yes	No	Yes	Yes	No	No	Yes
Fradet	2004	Canada	Yes	Yes	Yes	Yes	Yes	No	Yes	Yes
Tinzel	2004	Austria/Eur	Yes	Yes	Yes	Yes	Yes	Yes	Yes	Yes
Groskopf	2006	US	Yes	Yes	Yes	Yes	No	Unclear	No	Yes
van Gils	2007	Netherlands/Eur	Yes	Yes	Yes	Yes	Yes	Yes	Yes	Yes
van Gils	2007	Netherlands/Eur	Yes	Yes	Yes	Yes	Yes	Yes	Yes	Yes
Marks	2007	US, Canada	Yes	Yes	Yes	Yes	Yes	Yes	Yes	Yes
Deras	2008	US, Canada	Yes	Yes	Yes	Yes	Yes	Yes	Yes	Yes
Haese	2008	Europe	Yes	Yes	Yes	Yes	Yes	Unclear	Yes	Yes
Laxman	2008	US	Yes	Yes	Yes	Yes	No	Unclear	Yes	Yes
Ouyang	2009	US	Yes	Yes	Yes	Yes	Yes	Yes	Yes	Yes
Shappell	2009	US	Yes	Yes	No	Yes	No	Unclear	No	Yes
Wang	2009	US	Yes	Yes	Yes	Yes	Yes	Yes	Yes	Yes
Mearini	2009	Italy/Eur	Yes	Yes	Yes	Yes	No	Yes	No	Yes
Henderson	2010	UK/Eur	Yes	Yes	Yes	Yes	Yes	Yes	Yes	Yes
Aubin	2010	US	Yes	Yes	Yes	Yes	Yes	Yes	Yes	Yes
Auprich	2010	Europe	Yes	Yes	Yes	Yes	Yes	Yes	Yes	Yes
Morotel	2010	Spain/Eur	Yes	Yes	Yes	Yes	Yes	Yes	Yes	Yes
Nyberg	2010	Sweden/Eur	Yes	Yes	Yes	Yes	Yes	Yes	Yes	Yes
Roobol	2010	Netherlands/Eur	Yes	Yes	Yes	Yes	Yes	Unclear	Yes	Yes
Rigau	2010	Spain/Eur	Yes	Yes	Yes	Yes	Yes	Yes	Yes	Yes
Shen	2010	China/Asian	Yes	Yes	Yes	Yes	No	Unclear	Yes	Yes
Schilling	2010	Germany/Eur	Yes	Yes	Yes	Yes	Unclear	Yes	No	Yes
Rubio-Briones	2011	Spain/Eur	Yes	Yes	Yes	Yes	Yes	Unclear	Yes	Yes
Adam	2011	South Africa	Yes	Yes	Yes	Yes	Yes	Yes	Yes	Yes
Cao	2011	China/Asian	Yes	Yes	Unclear	Yes	No	Yes	No	Yes
Ochiai	2011	Japan	Yes	Yes	Yes	Yes	Yes	Unclear	Yes	Yes
Perdona	2011	Italy/Eur	Yes	Yes	Yes	Yes	Yes	Yes	Yes	Yes
Taille	2011	Europe	Yes	Yes	Yes	Yes	Yes	Yes	Yes	Yes
Babera	2012	Italy/Eur	Yes	Yes	Yes	Yes	Yes	Yes	Yes	No
CF Ng	2012	China/Asian	Yes	Yes	Yes	Yes	Yes	Yes	Yes	No
Crawford	2012	US	Yes	Yes	Yes	Yes	Yes	Yes	Yes	Yes
Pepe	2012	Italy/Eur	Yes	Yes	Yes	Yes	Yes	Yes	Yes	Yes
Pepe	2012	Italy/Eur	Yes	Yes	Yes	Yes	Yes	Yes	Yes	Yes
Sciarra	2012	Italy/Eur	Yes	Yes	Yes	Yes	Yes	Yes	Yes	Yes
Wu	2012	US	Yes	Yes	Yes	Yes	No	Yes	No	Yes
Ferro	2013	Europe	Yes	Yes	Yes	Yes	Yes	Yes	Yes	Yes
Goode	2013	US	Yes	Yes	Yes	Yes	No	Yes	No	Yes
Ochiai	2013	Japan	Yes	Yes	Yes	Yes	Yes	Unclear	Yes	Yes
Stephan	2013	Germany/Eur	Yes	Yes	Yes	Yes	Yes	Yes	Yes	Yes
Perdona‘	2013	Italy/EurCaucasian	Yes	Yes	Yes	Yes	Yes	Yes	Yes	Yes
Salagierski	2013	Poland/Eur	Yes	Yes	Yes	Yes	Yes	Yes	Yes	Yes
Scattoni	2013	Italy/Eur	Yes	Yes	Yes	Yes	Yes	Yes	Yes	Yes
Chevli	2013	USA	Yes	Yes	Yes	Yes	No	Yes	Yes	Yes
Giuseppe	2014	Italy/Eur	Yes	Yes	Yes	Yes	Yes	No	Yes	Yes
Francesco	2014	Italy/Eur	Yes	Yes	Yes	Yes	Yes	Yes	Yes	Yes

**Table 2:** PCA3 stratified analysis.

	Data sets	Sensitivity (95% CI)	Specificity (95% CI)	Diagnostic OR (95% CI)	I^2^, %*	AUC (95% CI)
Total	43					
Design						
Case-control	8	0.63 (0.61–0.66)	0.88 (0.87–0.90)	10.36 (5.51–21.25)	36.9	0.82 (0.79–0.85)
Prospective	39	0.65 (0.63–0.66)	0.73 (0.72–0.74)	5.31 (4.19–6.73)	70.5	0.75 (0.74–0.76)
biopsy						
Initial*	14	0.65 (0.63–0.67)	0.82 (0.81–0.83)	8.14 (4.78–13.86)	51.8	0.80 (0.78–0.82)
Repeated*	11	0.58 (0.55–0.62)	0.69 (0.67–0.71)	3.19 (2.62–3.83)	0	0.68 (0.67–0.70)
Mixed	22	0.66 (0.64–0.68)	0.68 (0.67–0.69)	5.13 (3.99–6.60)	76.9	0.75 (0.74–0.76)
cut-off value						
Equal 35	26	0.63 (0.62–0.65)	0.74 (0.73–0.75)	4.75 (3.42–6.60)	64.1	0.74 (0.73–0.76)
Not equal 35	21	0.70 (0.68–0.73)	0.67 (0.65–0.69)	6.22 (4.62–8.37)	62.6	0.77 (0.76–0.79)
